# Ontario Veterinary College

**Published:** 1885-04

**Authors:** 


### AMERICAN VETERINARY COLLEGE.

### ONTARIO VETERINARY COLLEGE.

On Thursday night, 26th February, at the semi-weekly meeting of the Veterinary Medical Society in connection with this College, the students presented to Prof. Smith, V.S., the much-esteemed Principal and President of the College, a picture of the Senior Graduating Class of 1884–5. The picture is four feet square. H. Piatt, St. Louis, Missouri, and D. E. McLean, Pilot Mound, Manitoba, in behalf of the students, presented the picture. G. McGillivray, Whitby, Ont., read the address, as follows:

To Prof. Smith, M.R.C.V.S., Principal of the Ontario Veterinary College.

*Dear Sir:* Since the commencement of our College course, the greatest of harmony has existed among us as students. Each of us being desirous of obtaining some memento of our college days, decided that a photo. of the class would be the most appropriate souvenir, and as a result obtained this picture, which we decided to present you as an appreciation of the hearty and sincere interest you take in our welfare, and the many kindnesses extended to us during our college course.

We take pleasure in presenting you this picture in behalf of the students whose photos. adorn this board, hoping there is no shadow on it, the original of which shall ever prove otherwise than a credit to the Ontario Veterinary College of which you are the worthy Principal.

Signed in behalf of the Senior Class,

G. McGillivray,<br>

D. E. McLean, } Committee.<br>

Harry Piatt,

Toronto, 26th Feb., 1885.

The Professor replied in an able and appropriate speech.

